# Supplementary material for: Observation of spontaneous spin-splitting in the band structure of an n-type zinc-blende ferromagnetic semiconductor
Source: Nat Commun. 2016 Dec 19;7:13810. doi: 10.1038/ncomms13810 (PMC5187429; doi:10.1038/ncomms13810)
Supplement: Supplementary Information — Supplementary Figures, Supplementary Notes and Supplementary References. [file ncomms13810-s1.pdf]

Supplementary Figures

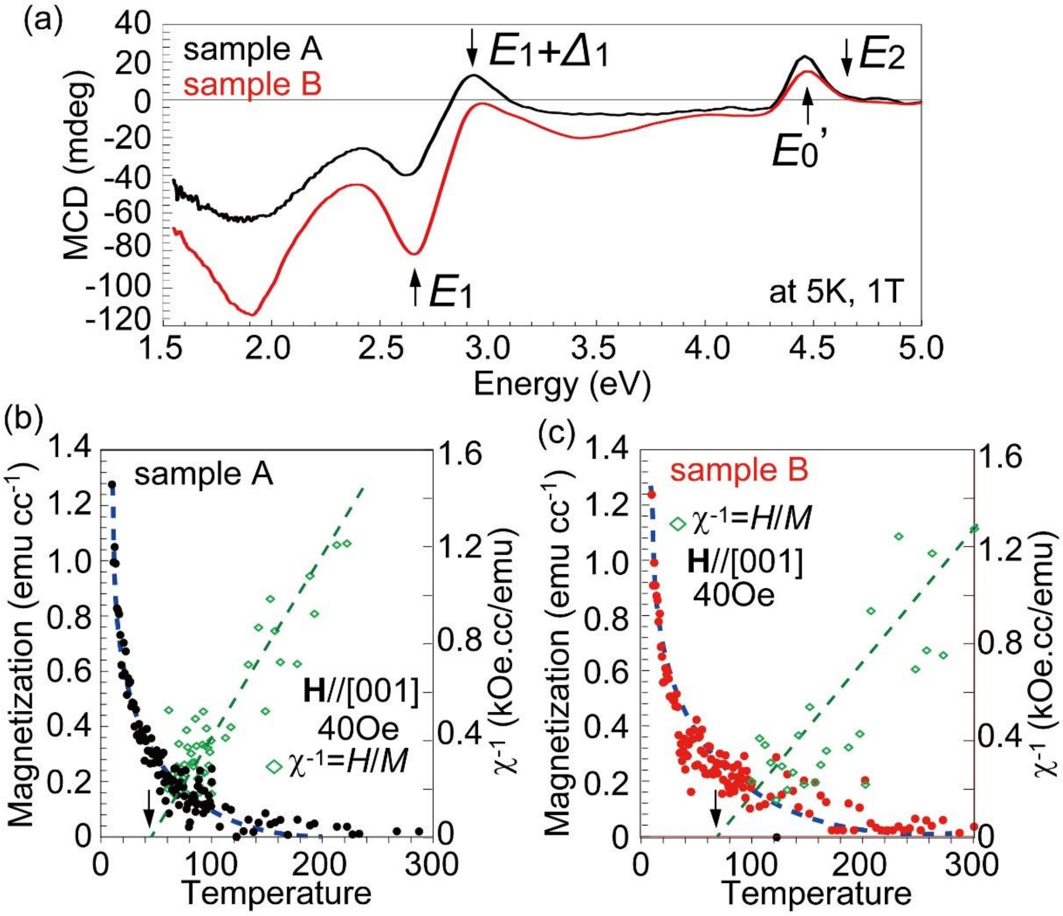

Supplementary Figure 1: Characterization of the magnetic properties. (a) MCD spectra of sample A (black curve) and sample B (red curve) measured at 5 K, under a perpendicular magnetic field of 1 T. (b) and (c) Temperature dependence of magnetization *M* (black or red circles, left axes) and inverse of the magnetic susceptibility χ (green open diamonds, right axes) of samples A and B, respectively, measured by SQUID. A small perpendicular magnetic field of 40 Oe was applied during the measurements. Blue dotted curves trace the *M*-*T* curves, serving as guides to the eye. Green dotted lines are the linear approximations of the χ^{-1} data, which roughly indicate the T_C values of 45 K for sample A and 65 K for sample B, as shown by black arrows.

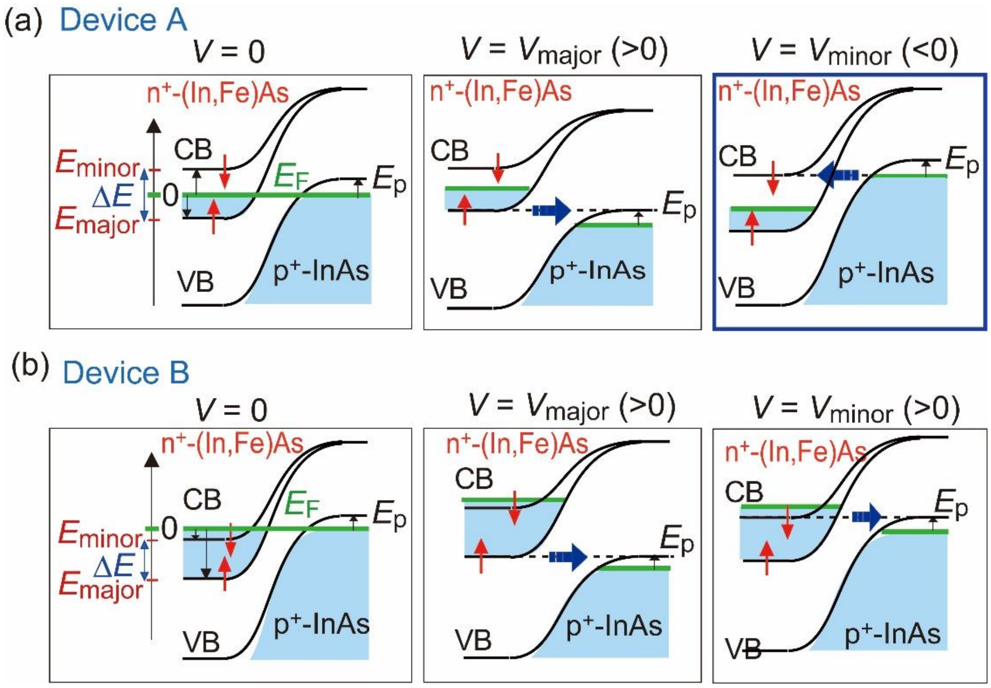

Supplementary Figure 2. Band profiles of the pn junctions in the two devices A and B. (a) and (b) are the band profiles of devices A and B, respectively, at bias voltage $V = 0$, V_{major}, and V_{minor}, all at low temperature (ferromagnetic state). At $V = 0$, with the Fermi energy at the zero point, the energy levels of the majority and minority spin CB bottom edges of (In,Fe)As and of the VB top of p^+-InAs are denoted as E_{major}, E_{minor}, and E_{p}, respectively. The blue arrows show the tunnelling directions of electrons at non-zero bias V. In device A, the tunnelling directions of electrons at $V = V_{\mathrm{major}}$ and at $V = V_{\mathrm{minor}}$ are opposite to each other.

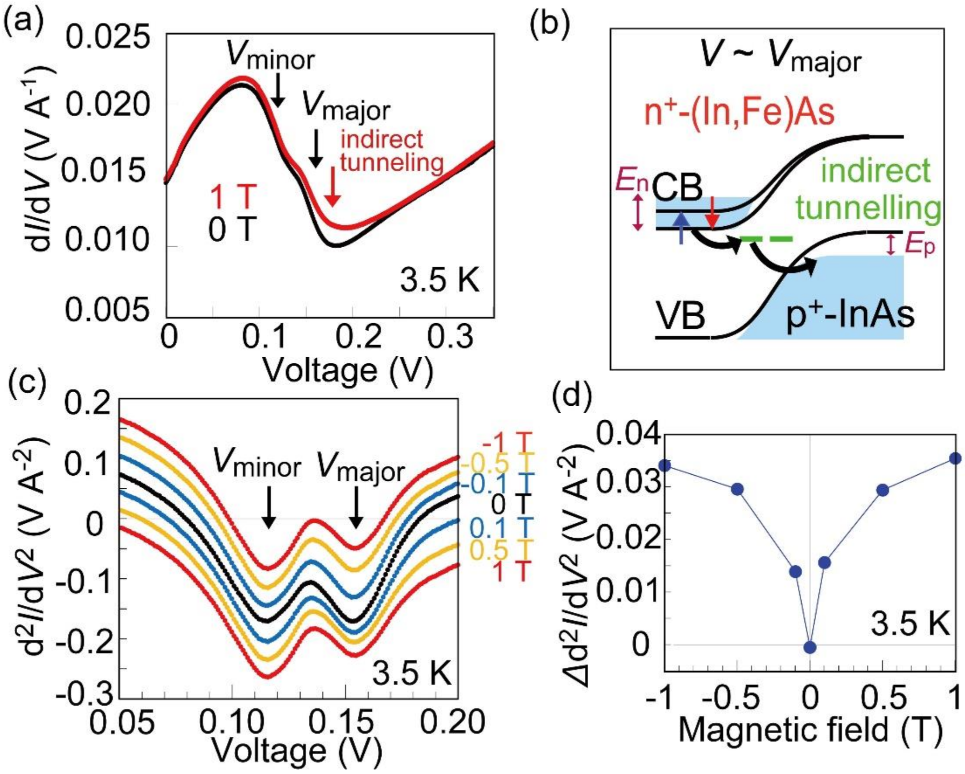

Supplementary Figure 3. Behavior of *I* – *V* curves of device B under various magnetic field strengths. (a) d*I*/d*V* – *V* curves of device B, measured at 3.5 K without and with a magnetic field *H* of 1 T applied in the film plane. At 1 T, d*I*/d*V* is increased at the end of the tunnelling region ($V \sim V_{major}$), which causes the shallowing of the majority spin valley in the d^2I/dV^2 – *V* curves at 1 T observed in the upper panel of Fig. 3c in the main manuscript. (b) Schematic energy diagram of the p-n junction at $V \sim V_{major}$. The enhancement of d*I*/d*V* is probably due to indirect tunnelling processes at the interface. (c) d^2I/dV^2 – *V* curves of device B at 3.5 K, measured under various magnetic fields *H* from -1 T to 1 T applied in the film plane. (d) Difference in the d^2I/dV^2 values of (c) at the majority and minority spin's valley centers, $\Delta d^2I/dV^2 = d^2I/dV^2(V_{major}) - d^2I/dV^2(V_{minor})$, as an indicator of the asymmetry between the two valleys under different *H*.

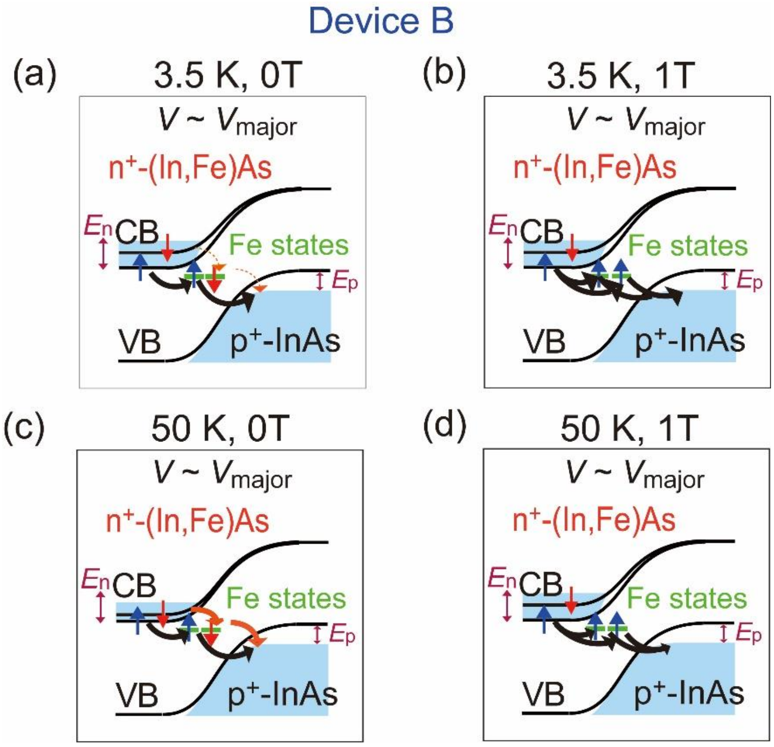

Supplementary Figure 4. Schematic energy diagram of the gap-state assisted tunnelling through paramagnetic Fe states (green lines) in device B. (a) and (b) are the situations at 3.5 K, without and with *H*, whereas (c) and (d) are the situations at 50 K, without and with *H*, respectively. At 3.5 K and 0 T (panel a) the gap-state assisted tunnelling is contributed mainly from the majority CB (blue arrow), whose energy is close to that of the Fe gap states. At 50 K and 0 T (panel c) both of the majority spin (blue arrow) and minority spin (red arrow) CBs contribute to the Fe gap-state assisted tunnelling. At 1 T (panels b and d), the spin angular momentums of the Fe gap states are aligned with the majority spin in the CB of (In,Fe)As, thus the tunnelling from the minority spin CB is prohibited, while the probability from the majority spin CB is enhanced.

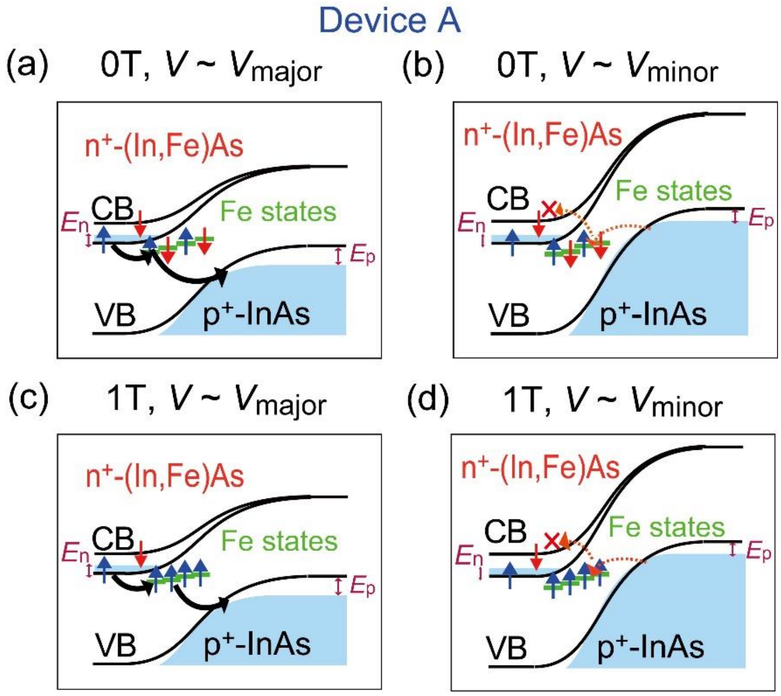

Supplementary Figure 5. Schematic energy diagrams of the gap-state assisted tunnelling through paramagnetic Fe states (green lines) in device A at 3.5 K. (a) and (b) are the situations at 0 T, at V_{major} and V_{minor}, whereas (c) and (d) are the situations at 1 T, at V_{major} and V_{minor}, respectively. The Fe gap state assisted tunnelling occurs at $V = V_{major}$, which is contributed by electrons in the majority spin CB, but is zero at $V = V_{minor}$. Due to the small electron density n of (In,Fe)As in device A (~1×10^{18} cm^{-3}), the depletion layer of the p-n junction extends more into the (In,Fe)As side, which increases the number of the Fe gap states and the indirect tunnelling current. At 1T (panel c, d) the spin angular momentums of the Fe gap states are aligned with the majority spin in the CB of (In,Fe)As. However, due to the small n of the (In,Fe)As layer, the indirect tunnelling current from the majority spin CB is almost unchanged.

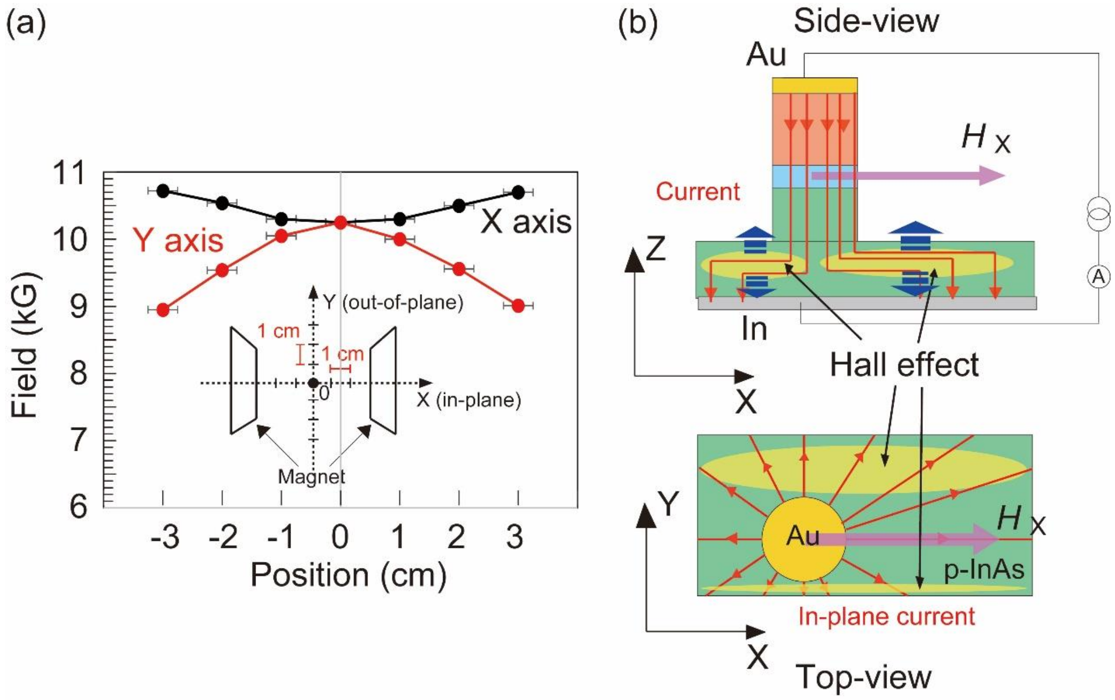

Supplementary Figure 6. Origin of the asymmetry in the d*I*/d*V* – *V* curves measured at +1T and -1T. (a) Magnetic field measured in the sample space between two poles of the electromagnet by a Gaussmeter, which confirmed the even-function symmetry of the magnetic field around the center position (inset). (b) Schematic sketch of the diode device placed in the magnetic field H_X, which is along the X direction. Red lines illustrate the current paths, which are mainly in the Z direction. However, in the p^+-InAs substrate (yellow areas) currents flow in the X-Y plane. The magnetic field H_X and the currents with Y-direction components induce Hall voltages in the Z direction. These Hall voltages, which are odd functions of the magnetic field, are not canceled out due to different path lengths of the currents in the substrate, thus can be detected by the top and bottom electrodes.

Supplementary Note 1:
Characterization of the magnetic properties

The magnetic properties of the two samples A and B were characterized by magnetic circular dichroism (MCD) in a reflection configuration and superconducting quantum interference device (SQUID) measurements.

Supplementary Figure 1(a) shows MCD spectra of samples A (black curve) and B (red curve), measured at 5 K and under 1 T applied perpendicular to the film plane. The MCD spectra of both samples show peaks corresponding to the optical transition at the critical points of InAs zinc blende (ZB) band structure: E_1 (2.61 eV), $E_1+\Delta_1$ (2.88 eV), E_0' (4.39 eV), and E_2 (4.74 eV)[1]. The MCD peak at lower energy (~2eV) is usually observed in bulk-like thick (In,Fe)As samples, although its origin is not clear at this stage. The MCD spectra confirmed the ZB-type crystal structure and band structure of (In,Fe)As in the two samples. One can also see that the MCD intensity of sample B (Fe content: 8%) is twice as large as that of sample A (Fe content: 6%), reflecting the different magnetization in the two samples.

Supplementary Figures 1(b) and (c) show the temperature dependence of magnetization (*M*-*T* curves) of the two samples A and B, respectively, measured by SQUID when increasing temperature from 10 K to 300 K. These are field cooling (FC) curves; before the *M*-*T* measurements, the samples were cooled to 10 K with a magnetic field of 1 T applied perpendicular to the film plane. Note that because of the small remanent magnetization in both samples, a weak perpendicular magnetic field of 40 Oe was applied during these *M*-*T* measurements which caused a tail up to temperatures above T_C. One can see that the magnetization of sample B persisted at high temperatures (100 K < T < 200 K), while that of sample A decreased quickly and was almost unchanged above 100 K. The inverse of the magnetic susceptibility $\chi^{-1} = H/M$ [at H = 40 Oe, T > 100 K, green open diamonds] follows the Curie-Weiss law $\chi = C/(T-T_C)$ [green dotted line, T_C is the Curie temperature, C is the Curie constant], allowing us to estimate T_C of the two samples. Although the χ^{-1} data points are fluctuating due to the low S/N ratio at high T, we can roughly estimate the T_C to be 45 K for sample A and 65 K for sample B, respectively.

Supplementary Note 2:
Estimation of the spin split energy ΔE of (In,Fe)As from the center positions of the two-valley structures in the $d^2I/dV^2 - V$ curves

The two spin-Esaki diode devices (A and B) studied in this work differ in Fe concentration (6% and 8%, respectively) and electron density (due to co-doping of Be double donors in the (In,Fe)As layer in device B). Both of the two diode devices show two-valley structures in the $d^2I/dV^2 - V$ curves (Figs. 2a-d and Figs. 3a-d in the main manuscript), corresponding to the splitting of the majority spin conduction band (CB) and minority spin CB of (In,Fe)As. For the two-valley structures, we fitted the sum of two Lorentzian curves to determine the valley center positions of the majority and minority spin CBs (V_{major} and V_{minor}, respectively) (see main manuscript). The spin split energy ΔE of the (In,Fe)As layers was estimated by the difference between V_{major} and V_{minor} as follows.

Supplementary Figures 2a and b show the band profiles of the p-n junctions in the two devices A and B, respectively, at low temperature (ferromagnetic state) and bias voltage $V = 0$, V_{major}, and V_{minor}. In these figures, we set the Fermi energy at the zero point, and denote the energy levels of the majority and minority spin CB bottom edges of (In,Fe)As and the valence band (VB) top of p^+-InAs as E_{major}, E_{minor}, and E_{p}, respectively. The spin split energy ΔE of (In,Fe)As is therefore given by $E_{\mathrm{minor}} - E_{\mathrm{major}}$.

In device A, because V_{major} is positive whereas V_{minor} is negative (data shown in Figs. 2a,b of the main manuscript), the Fermi level E_{F} lies above the band edge of the majority spin CB and below that of the minority spin CB ("half-metallic" band structure, i.e. $E_{\mathrm{major}} < 0$, $E_{\mathrm{minor}} > 0$), as illustrated in Supplementary Figure 2a. At V_{major}, the band edge of majority spin CB of (In,Fe)As is aligned with the top of the VB of p^+-InAs, thus $eV_{\mathrm{major}} = -E_{\mathrm{major}} + E_{\mathrm{p}}$. However, at V_{minor}, which is negative, the band edge of the minority spin CB of (In,Fe)As is aligned with the quasi-Fermi level of the p^+-InAs, thus $eV_{\mathrm{minor}} = -E_{\mathrm{minor}}$. Therefore, we have the following relation:

$$e(V_{\mathrm{major}} - V_{\mathrm{minor}}) = E_{\mathrm{minor}} - E_{\mathrm{major}} + E_{\mathrm{p}} = \Delta E + E_{\mathrm{p}} \qquad (1)$$

This means that the difference between V_{major} and V_{minor} overestimates the spin split energy ΔE of (In,Fe)As CB in device A by E_{p}. To correct this overestimation, we have subtracted $e(V_{\mathrm{major}} - V_{\mathrm{minor}})$ of device A by E_{p}, which is estimated to be 8.3 meV from the Be doping concentration of 1×10^{18} cm^{-3}.

On the other hand, in device B, because both V_{major} and V_{minor} are positive (data shown

in Figs. 2c,d of the main manuscript), the Fermi level E_F in (In,Fe)As lies above both the majority and minority spin CB bottom edges as illustrated in Supplementary Figure 2b (thus, $E_{major} < 0$ and $E_{minor} < 0$). At V_{major} (V_{minor}), the band edge of the majority (minority) spin CB of (In,Fe)As is aligned with the top of the VB of p^+-InAs. Therefore we have the following relations:

$$eV_{major} = -E_{major} + E_p \tag{2}$$

$$eV_{minor} = -E_{minor} + E_p \tag{3}$$

$$e(V_{major} - V_{minor}) = E_{minor} - E_{major} = \Delta E \tag{4}$$

Thus the difference between V_{major} and V_{minor} corresponds exactly to the spin split energy ΔE of (In,Fe)As CB in device B.

Supplementary Note 3:
Behavior of the majority and minority spin's valleys in the $d^2I/dV^2 – V$ curves of the two devices under various magnetic fields

From the upper panels of Figs. 3a, c, and d (the minority and majority spin Lorentzian curves of each experimental data), one can see that in Figs. 3a and d, the majority spin valley is smaller (shallower) than the minority spin valley at zero magnetic field, whereas in Fig. 3c the majority spin valley became shallower with applying **H**. In the following, we explain possible reasons for this complicated behavior.

First, we show in Supplementary Figure 3a the $dI/dV – V$ curves of device B at 3.5 K and under 0 T (black) and 1 T (red). One can see that the shallowing of the majority spin valley in the $d^2I/dV^2 – V$ curve is caused by the increase of the dI/dV after the end of the direct tunnelling region ($V \sim V_{major}$) of the Esaki diode (indicated by the red arrow in Supplementary Figure 3a). As illustrated in Supplementary Figure 3b, at the end of the tunnelling region ($V \sim V_{major}$), the (In,Fe)As conduction band (CB) bottom (majority spin CB bottom) is lifted to the same energy as the p^+-InAs valence band (VB) top, and direct tunnelling from CB to VB is suppressed. Therefore, the increase of the dI/dV after the end of the tunnelling region reflects the tunnelling conductance due to other indirect tunnelling processes, such as magnon-assisted tunnelling, phonon-assisted tunnelling, gap-state assisted tunneling, or their combinations.

In Supplementary Figure 3c, we plot $d^2I/dV^2 – V$ curves of device B, measured at 3.5 K under various magnetic field strengths H from -1 T to 1 T applied in the film plane (the data at 0 T and 1 T are the same as those plotted in Fig. 3c). To show the dependence of the asymmetry between the majority and minority spin's valleys on the magnetic field

strength H, we plot in Supplementary Figure 3d the difference in the d^2I/dV^2 values at the majority and minority spin's valley center, $\Delta d^2I/dV^2 = d^2I/dV^2(V_{major}) - d^2I/dV^2(V_{minor})$, as a function of the magnetic field H. We see that $\Delta d^2I/dV^2 - H$ shows the same nonlinear behavior under positive and negative H. This result indicates that the indirect tunnelling process in device B is magnetic-field dependent.

Here, we propose a scenario of *gap-state assisted tunnelling through paramagnetic Fe-induced gap states* at the interface of the p-n junction: At the interface or in the depletion region of the p-n junction, some Fe gap states can exist due to diffusion of Fe atoms from the (In,Fe)As electrode. The energy levels of these paramagnetic Fe-induced states are close to the CB bottom of (In,Fe)As [2]. Thus, electrons at the CB bottom of (In,Fe)As can indirectly tunnel to the VB top of p^+-InAs through these paramagnetic Fe gap states after the end of the direct tunneling region.

Supplementary Figures 4 and 5 illustrate the schematic energy diagrams of the paramagnetic Fe gap states in devices A and B, respectively, at different temperatures and magnetic fields. Using these diagrams, we will explain the behavior of the two spin valleys in the $d^2I/dV^2 - V$ curves in Figs. 3a, c, and d, as follows.

Behavior of the $d^2I/dV^2 - V$ curves in Fig. 3c (device B, 3.5 K)

At 3.5 K and 0 T (Supplementary Figure 4a), the Fe gap-state assisted tunnelling occurs mainly from the majority spin CB, whose energy is close to that of the Fe gap states, to the p^+-InAs VB through the paramagnetic Fe gap states that have the same spin magnetic moment direction (blue arrows in Supplementary Figure 4a).

At 3.5 K and 1 T (Supplementary Figure 4b), however, there are more Fe gap states whose magnetic moments aligned with the majority spins in the CB of (In,Fe)As. Thus, indirect tunnelling from the majority spin CB is enhanced. This explains the increase of the dI/dV after the end of the direct tunneling region (Supplementary Figure 3a) and the shallowing of the majority spin valley of the $d^2I/dV^2 - V$ curves (Fig. 3c) when H was applied.

Behavior of the $d^2I/dV^2 - V$ curves in Fig. 3d (device B, 50 K)

In the upper panel of Fig. 3d, the majority spin valley is shallower than the minority spin valley at 0 T, and becomes slightly deeper when applying H, although it is still shallower than the minority spin valley. We can explain this behavior as follows. At 50 K, electrons from both the majority spin (blue arrow) and minority spin (red arrow) CBs can tunnel through the paramagnetic Fe gap states and contribute to the indirect tunnelling current, as shown in Supplementary Figure 4c. This is possible because of the

smaller spin split energy of (In,Fe)As CB and phonon-assisted processes existing at 50 K. This explains why the majority spin valley at 50 K (Fig. 3d) is shallower than that at 3.5 K (Fig. 3c) at 0 T. However, when H was applied, more magnetic moments of the paramagnetic Fe states are aligned with the majority spin in the CB of (In,Fe)As, and the indirect tunnelling from the minority spin CB is partly suppressed, as shown in Supplementary Figure 4d. Therefore the total indirect tunnelling current decreases, which explains why the majority spin valley of the $d^2I/dV^2 - V$ curve at 50 K in devices B becomes less shallow (slightly deeper) with applying H as seen in Fig. 3d.

Behavior of the $d^2I/dV^2 - V$ curves in Fig. 3a (device A, 3.5 K)

In the upper panel of Fig. 3a in the main manuscript, the majority spin valley is shallower than the minority spin valley even at 0 T, and the two spin valleys change very little with H. Supplementary Figure 5 shows the schematic energy diagrams of the gap-state assisted tunnelling through paramagnetic Fe states (green lines) in device A at bias voltages $V = V_{major}$ and $V = V_{minor}$. In device A, because the Fermi level of (In,Fe)As lies above the bottom of the majority spin CB but below that of the minority spin CB, the tunneling direction of electrons at $V = V_{minor}$ is opposite to that at $V = V_{major}$, as illustrated in Supplementary Figure 5 (see also Supplementary Figure 2). At 0 T and $V = V_{major}$ (Supplementary Figure 5a), the Fe gap-state assisted tunnelling current is contributed only by electrons in the majority spin CB (blue arrow) of (In,Fe)As, because the minority spin CB (red arrow) is empty. Meanwhile, at $V = V_{minor}$ (Supplementary Figures 5b,d) the minority spin electrons in the VB of p^+-InAs, however, cannot tunnel into the minority spin CB of (In,Fe)As through the Fe gap states because the energy levels of the Fe gap states are lower than the minority CB bottom edge. Therefore the Fe gap-state assisted tunnelling current at $V = V_{minor}$ is zero. This difference of the Fe gap-state assisted tunnelling currents in the cases of $V = V_{major}$ and $V = V_{minor}$ explains why the majority spin valley is shallower than the minority spin valley at 0 T. We also note that because the electron density n of (In,Fe)As is lower ($\sim 1 \times 10^{18}$ cm^{-3}) than device B, the depletion layer of the p-n junction extends more into the (In,Fe)As side. Due to the lack of carriers inside the depletion region of (In,Fe)As, more Fe atoms act as paramagnetic Fe states, which increases the number of the Fe gap states. This situation further enhances the Fe gap-state assisted tunnelling current at $V = V_{major}$ in device A in comparison with that of device B.

When applying H = 1 T (Supplementary Figure 5c), the number of majority spin Fe gap states increases. However, due to the small electron density n in the CB of (In,Fe)As layer, an increase in the number of majority spin Fe gap states (which is

already quite large at 0 T) does not yield any large effect. This is why the gap-state assisted tunnelling current shows almost no change. Besides, other magnetic-field-independent mechanism (ex. phonon-assisted tunnelling) may be dominant in device A.

Supplementary Note 4:
Origin of the asymmetry between the d*I*/d*V* – *V* curves of device A measured at +1 T and -1 T

In the TAMR measurements, the external magnetic field **H** was kept fixed at 1 T and rotated in the film plane, and the d*I*/d*V*-*V* curves of device A were measured at various **H** directions with every step of 10 degrees at 3.5 K. At each direction of **H**, we noticed that the d*I*/d*V*-*V* curves measured at the magnetic field of 1 T and -1 T are slightly different. The difference in the d*I*/d*V* – *V* curves of 1T and -1T is caused by an *odd* function contribution of the magnetic field **H**, due to the Hall effect occurring in the p^+-InAs substrate, as explained below.

The devices were placed meticulously in the center position of the space between the two poles of our electromagnet (misalignment, if any, should be in millimeter order). In principle, with the center position as the origin, the distribution of magnetic field in this sample space is an even function of the position. Supplementary Figure 6a show the position dependence of **H** in the sample space of our electromagnet measured by a Gaussmeter, which confirmed the even-function symmetry of the magnetic field. Therefore misalignment of the sample from the center position, if any, cannot generate a response that is an odd function of **H**.

In Supplementary Figure 6b, we show a general situation when the sample is placed in the X-Y plane under a magnetic field *H* applied in the X direction (H_X). The currents (red lines) flow through the mesa diode in the Z direction. In the thick p^+-InAs substrate, however, the currents can flow in the X-Y plane (yellow areas in Supplementary Figure 6b). Because the top mesa is not located exactly at the center of the substrate, different path lengths are expected for currents flowing in different directions in the X-Y plane (see the top-view in Supplementary Figure 6b). In these yellow areas, the magnetic field H_X and the currents in the Y-direction induce Hall voltages in the Z direction. These Hall voltages are not canceled out due to different path lengths of the currents in the substrate, thus can be detected by the top and bottom electrodes. These Hall voltages are odd functions of the magnetic field and thus have opposite signs at +1 T and -1 T. We think that this is the main origin of the difference in the d*I*/d*V* – *V* data under +1 T and

-1 T.

Supplementary References

1. Hai, P. N., *et al.*, Growth and characterization of n-type electron-induced ferromagnetic semiconductor (In,Fe)As. *Appl. Phys. Lett.* **101,** 182403 (2012).
2. Huang, K., Wessels, B. W., Electronic and optical properties of deep levels in iron‐doped InAsP alloys. *J. Appl. Phys.* **64**, 6770-6774 (1988).
